# Excision and transfer of an integrating and conjugative element in a bacterial species with high recombination efficiency

**DOI:** 10.1038/s41598-019-45429-z

**Published:** 2019-06-20

**Authors:** Evelyn Weiss, Carolin Spicher, Rainer Haas, Wolfgang Fischer

**Affiliations:** Max von Pettenkofer Institute of Hygiene and Medical Microbiology, Faculty of Medicine, LMU München, 80336 München, Germany

**Keywords:** Mobile elements, Bacterial genetics

## Abstract

Horizontal transfer of mobile genetic elements, such as integrating and conjugative elements (ICEs), plays an important role in generating diversity and maintaining comprehensive pan-genomes in bacterial populations. The human gastric pathogen *Helicobacter pylori*, which is known for its extreme genetic diversity, possesses highly efficient transformation and recombination systems to achieve this diversity, but it is unclear to what extent these systems influence ICE physiology. In this study, we have examined the excision/integration and horizontal transfer characteristics of an ICE (termed ICE*Hptfs4*) in these bacteria. We show that transfer of ICE*Hptfs4* DNA during mating between donor and recipient strains is independent of its conjugation genes, and that homologous recombination is much more efficient than site-specific integration into the recipient chromosome. Nevertheless, ICE*Hptfs4* excision by site-specific recombination occurs permanently in a subpopulation of cells and involves relocation of a circularization-dependent promoter. Selection experiments for excision indicate that the circular form of ICE*Hptfs4* is not replicative, but readily reintegrates by site-specific recombination. Thus, although ICE*Hptfs4* harbours all essential transfer genes, and typical ICE functions such as site-specific integration are active in *H*. *pylori*, canonical ICE transfer is subordinate to the more efficient general DNA uptake and homologous recombination machineries in these bacteria.

## Introduction

Integrating and conjugative elements (ICEs) are mobile genetic elements carrying a wide range of cargo genes which confer upon their hosts traits such as additional metabolic or colonization abilities, or antibiotic or metal resistance^1^. They are typically integrated into the host chromosome and get replicated and transferred to daughter cells during cell division, but they can also excise from the host chromosome and spread horizontally by conjugative transfer to a recipient cell. Subsequently, transferred circular ICE intermediates can integrate into the recipient cell’s chromosome, thus completing a canonical ICE life cycle^1,2^. ICEs have been identified in a broad range of bacterial species^3^, but many of these systems have not been characterized in detail.

The human gastric pathogen *H*. *pylori* is a bacterial species with an extremely high genetic diversity due to elevated mutation and recombination rates, and this diversity is thought to enable adaptation to changing environments during chronic infection^4^. It is not uncommon that individuals are co-infected with two or more distinct strains. Therefore, *H*. *pylori* genomes evolve during long-term infection not only by accumulating mutations, but also by import and recombination of larger DNA fragments from co-infecting strains, which may amount to significant fractions of the corresponding genomes, making recombination the most important driver of diversification^5,6^. DNA uptake is mediated by a natural transformation system, comprising the ComB type IV protein transport system and the ComEC cytoplasmic membrane DNA channel^7,8^, which reaches enormous uptake rates^9^.

Apart from uptake and homologous recombination of extracellular DNA fragments, mobile genetic elements, such as plasmids^10^, phages^11^ and ICEs^12^ may contribute to increased strain diversity. The *H*. *pylori* ICEs (ICE*Hptfs3* and ICE*Hptfs4*), which are sometimes located in regions of genome plasticity, but have a variety of possible integration sites^12,13^, generally contain complete sets of genes required to produce type IV secretion machineries, as well as genes encoding different DNA-processing proteins^13–15^. Several ICE genes have been suggested as disease markers, for instance *dupA* as a marker for duodenal ulcer^16,17^, or *jhp950* as a marker for marginal zone B-cell MALT lymphoma^18^. Moreover, virulence-associated phenotypes of individual ICE-encoded proteins have been described^19,20^, but the functions of the ICEs within the infection process are currently not well-understood.

We have previously reported that a resistance gene marker inserted into the ICE*Hptfs4* element of *H*. *pylori* strain P12 could be transferred from donor to recipient bacteria co-cultivated in the presence of DNase I to prevent natural transformation, suggesting that conjugative transfer of the whole genome island takes place^13^. However, such a transfer was only achieved when recipient strains harboring at least a part of a similar ICE element were used. Here, we have characterized this transfer as well as the excision step from the chromosome further, and we show that none of the genes on ICE*Hptfs4* is essential for transfer of the resistance marker under standard mating conditions. We have further characterized the requirements for excision of ICE*Hptfs4* from the chromosome, and we show that transfer of this 41 kb ICE is independent of the presence of integration sites or the XerT recombinase. Thus, although comparative genomic analysis strongly suggests a transfer activity of ICE*Hptfs4* in a classical, ICE-like manner, this mechanism is superimposed in *H*. *pylori* by the much more efficient recombination machinery.

## Results

### Horizontal transfer of ICE*Hptfs4* fragments is mediated by homologous recombination

**Figure 1:**
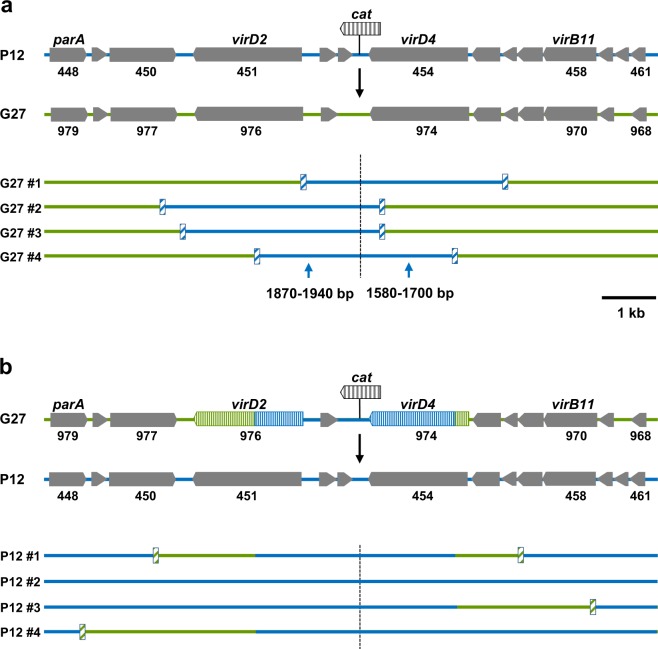
Determination of transferred fragment sizes in a homeologous system. (**a**) Mating experiments were performed using a P12 donor strain with a *cat* resistance gene inserted between ICE*Hptfs4* genes *hpp12_453* and *hpp12_454*, and a kanamycin-resistant G27 recipient strain. Genes flanking the *cat* cassette in P12, and orthologous genes from G27, are indicated by numbers. Flanking regions were sequenced in the resulting double-resistant transconjugant clones, and alignment with P12 and G27 sequences, respectively, was used to determine regions in the G27 recipient genome (green lines) that had been replaced by P12 donor strain DNA (blue lines). Four representative clones are shown; the position of the *cat* cassette is indicated by a broken line, and approximate recombination breakpoints by hatched boxes. (**b**) For a reverse experiment, DNA of transconjugant #4 of panel A was reintroduced into G27 wild-type by selection for chloramphenicol resistance, and a *recA* deletion was introduced subsequently. The resulting strain was used as a donor strain and mated with a kanamycin-resistant P12 recipient. Recombination breakpoints of representative transconjugant clones are indicated as in (A); for transconjugant clones where recombination had taken place within the P12 sequence region in the donor strain genome, breakpoints could not be determined.

ICEs are generally transferred horizontally by conjugation of circular intermediates excised from the chromosome^2^. When ICE*Hptfs4* was provided with a chloramphenicol resistance (*cat*) gene, transfer of this resistance could be shown by co-cultivation with a recipient strain in the presence of DNase I^13^. However, since heterologous recipient strains lacking ICE*Hptfs4* failed to take up the resistance gene in such transfer experiments, we were previously unable to show transfer of the complete ICE. Thus, we could not exclude partial ICE transfer and integration via homologous recombination rather than site-specific integration. To be able to estimate the size of transferred DNA segments, we first performed transfer experiments in a homeologous system, i.e. with the P12 [ICE*Hptfs4::cat*] donor and a kanamycin-resistant G27 recipient strain. Individual double-resistant clones were analysed by sequencing the region surrounding the insertion site of the *cat* gene, and single-nucleotide polymorphisms between donor and recipient strains were used to identify recombination breakpoint regions. Such breakpoints were typically located 400–4,000 bp away from the *cat* insertion site (Fig. 1a). Since these import lengths are typical for DNA introduced by natural transformation and subsequent homologous recombination^21–23^, this indicates that most transfer events under the applied conditions are indeed mediated by homologous recombination into a pre-existing ICE, rather than by transfer and site-specific integration of the complete element. To check for strain dependence, we conducted a reverse mating experiment with a chloramphenicol-resistant G27 donor and a kanamycin-resistant P12 recipient, and found similar, or slightly elevated, import lengths that ranged up to 13500 bp (Fig. 1b). Such higher import lengths (>13 kb) were also detected previously for transfer experiments between homologous strains^13^, and were interpreted as transfer of the complete island, but the present data suggest that an exchange of smaller ICE fragments occurs preferentially.

### Transfer of full-length ICE*Hptfs4* occurs at low frequency independent of the *tfs4* type IV secretion system

**Figure 2:**
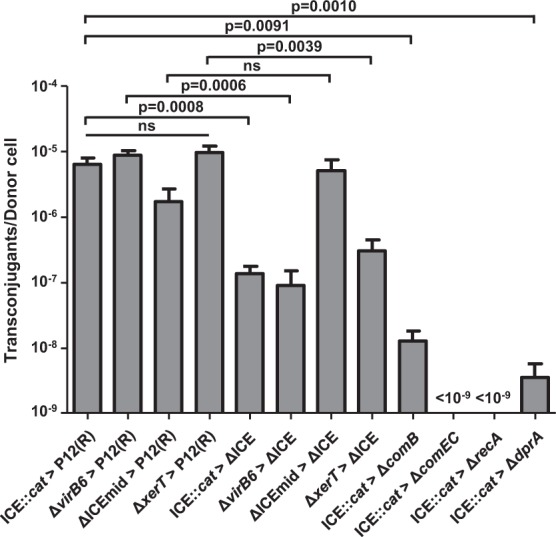
Transfer rates of ICE*Hptfs4* to ICE-positive and ICE-negative recipients. Mating experiments in the presence of DNase I were performed between the ICE*Hptfs4::cat* donor strain (as shown in Fig. 1a; ICE*::cat*), or isogenic donor strains with complete deletions of the indicated genes, and either a kanamycin-resistant P12 wild-type strain (P12(R)), or an ICE-deficient P12 mutant (ΔICE) as recipients. The ΔICEmid donor strain has a deletion of genes *hpp12_438* to *hpp12_469* (see Fig. S1). Likewise, isogenic mutants in genes encoding DNA uptake factors (Δ*comB*, Δ*comEC*) or recombination proteins (Δ*recA*, Δ*dprA*) were provided with a kanamycin resistance and used as recipient strains. Transfer rates are indicated as double-resistant clones per viable donor cell; no clones were obtained with Δ*comEC* and Δ*recA* recipients. Viability of recipient cells was confirmed by plating co-cultivated bacteria in serial dilutions on kanamycin-containing media. All data shown are mean values of at least five independent experiments with standard errors of the mean (number of experiments from left to right: n = 16, 9, 7, 5, 14, 6, 5, 6, 7, 13, 9 and 13); individual p values are indicated above the bars; ns, no significant difference.

To investigate whether ICE*Hptfs4* can be transferred entirely under our experimental conditions as well, we examined isogenic ICE deletion mutants as recipient strains. Direct deletion of full-length ICE*Hptfs4* from strain P12 was not possible, whereas replacing an internal part of ICE*Hptfs4* (genes *hpp12_438* to *hpp12_469*) with a chloramphenicol or erythromycin resistance cassette was (Fig. S1; ΔICEmid). Subsequent transformation with an ICE*Hptfs4* deletion plasmid (Fig. S1; ΔICE) resulted in a situation as in the corresponding regions of ICE-free strains^12^, where one of the integration sites is retained. Next, we provided the ICE*Hptfs4* deletion mutant with a kanamycin resistance (Δ*moeB::aphA*-3), and used it for mating experiments. Transfer from the donor strain P12 [ICE*Hptfs4::cat*] to this deletion mutant was still possible, although rates were reduced by almost two orders of magnitude in comparison to those obtained with a ICE*Hptfs4*-positive recipient (1.35 vs. 63.9 × 10^−7^ transconjugants per donor cell; Fig. 2).

Since ICE*Hptfs4* contains orthologs of all essential conjugation apparatus genes (the *tfs4* genes), and also a relaxase and a coupling protein gene^13^, we reasoned that this conjugation system might be responsible for ICE*Hptfs4* transfer. However, when we used donor strains with a deletion of the *virB6* ortholog, or the *xerT* gene encoding a site-specific recombinase involved in ICE*Hptfs4* excision^13^, we did not see a reduction in transfer rates (Fig. 2). In fact, when a mutant lacking all putative type IV secretion genes (P12ΔICEmid; Fig. S1), was used as a donor strain, it was still able to transfer its chloramphenicol resistance to a wild-type recipient at a slightly reduced rate, which did not reach statistical significance (Fig. 2). When an ICE*Hptfs4*-free mutant was used as a recipient strain, transfer rates from the *virB6* and *xerT* donor strains were reduced to a similar degree as with the ICE*Hptfs4::cat* donor. In contrast, the P12ΔICEmid donor strain even showed a slightly higher transfer rate into the ΔICE*Hptfs4* than into the wild-type recipient (Fig. 2), indicating that this short truncated element integrates preferentially by homologous recombination, as shown above in the homeologous system. Collectively, these observations suggest that none of the putative *tfs4* type IV secretion components is essential for ICE*Hptfs4* transfer.

### DNA uptake and recombination proteins are essential for ICE*Hptfs4* transfer in the recipient strain

We have previously shown that conjugative transfer of *H*. *pylori* plasmids is enhanced by an active competence type IV secretion (ComB) system in the recipient^24^. To characterize horizontal transfer of ICE*Hptfs4* further, we therefore performed mating experiments with recipient strains lacking genes required for DNA uptake or processing. DNA uptake is considered a two-step process, in which transport across the outer membrane is mediated by the ComB system^7,8^, and transport across the cytoplasmic membrane by the ComEC channel protein^8,25^. Interestingly, when we used a Δ*comB* recipient strain for transfer experiments, we obtained ICE*Hptfs4* transconjugants at a strongly reduced rate (Fig. 2), suggesting that the ComB apparatus is not strictly required, but supports DNA uptake during ICE*Hptfs4* transfer, as previously reported for plasmid DNA^24^. To our surprise, however, no transconjugants were ever obtained with a Δ*comEC* recipient strain, indicating that ComEC is strictly required not only for DNA uptake by transformation^8,26^, but also during conjugation. During transformation, DNA taken up is integrated into the chromosome via homologous recombination, which is driven by the RecA recombinase and supported by the loading protein DprA^27^. To check whether chromosomal integration of ICE*Hptfs4* occurs independent of homologous recombination, we therefore used Δ*recA* and Δ*dprA* mutant strains as recipients. Surprisingly, the Δ*recA* mutant was unable to integrate ICE*Hptfs4* DNA, while integration was still possible in the *dprA* mutant, albeit at minimal rates (Fig. 2). Transconjugants obtained in the Δ*comB* and the Δ*dprA* backgrounds were confirmed by PCR and sequencing for correct transfer of the *cat* gene (data not shown). These data suggest either that site-specific integration of ICE*Hptfs4* involves a process for which RecA is strictly required, or that integration does not occur site-specifically, but by homologous recombination.

### Excision of ICE*Hptfs4* from the chromosome involves relocation of a promoter

**Figure 3:**
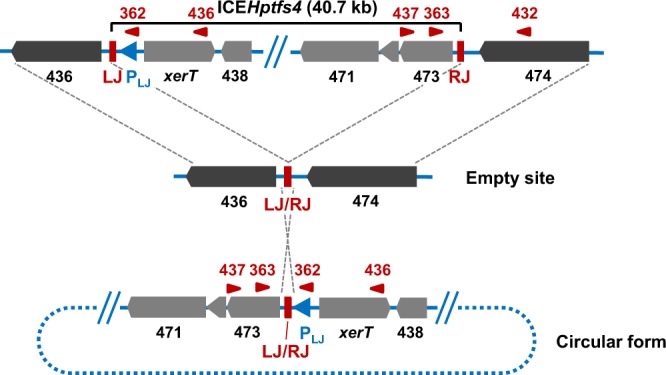
Rearrangement of ICE*Hptfs4* genetic elements upon excision from the genome. Schematic representation of ICE*Hptfs4* excision, which results in juxtaposition of the left and right junctions (LJ/RJ). This rearrangement can be detected by primers (red arrowheads) such as WS362/WS363 or WS436/WS437, while the integrated ICE is detected with primers WS363 and WS432. A putative outward-facing promoter (P_LJ_) which relocates upstream of *hpp12_473* upon excision is indicated by a blue arrowhead. Other promoters, such as the *xerT* promoter, which is still oriented towards *xerT* in the circular form, are not shown.

To address the question whether site-specific recombination plays a role for ICE*Hptfs4* transfer, we decided to characterize ICE excision further. We have demonstrated previously that excision of ICE*Hptfs4* from the chromosome to form a circular product (Fig. 3) depends on the XerT recombinase^13^. However, a nested PCR was necessary to demonstrate this circular product, whereas PCR with a single primer pair did not result in visible products, suggesting that excision is not very frequent. In all *H*. *pylori* genomes containing non-truncated ICE*Hptfs4* elements, the border regions show a well-conserved arrangement with 400 bp upstream of the *xerT* orthologs and only about 55 bp upstream of the *hpp12_473* orthologs^12^. Interestingly, primary transcriptome analysis in strain 26695 identified no transcriptional start site upstream of the *hpp12_473* ortholog *hp446*, but two transcriptional start sites upstream of the ICE*Hptfs4b xerT* ortholog *hp1009*, one of which is directed towards the ICE*Hptfs4b* left junction (LJ)^28^. Supposing that such a promoter exists in ICE*Hptfs4* of strain P12 as well, despite only low sequence similarity, this promoter would become oriented upstream of *hpp12_473* upon ICE excision from the chromosome (Fig. 3).

**Figure 4:**
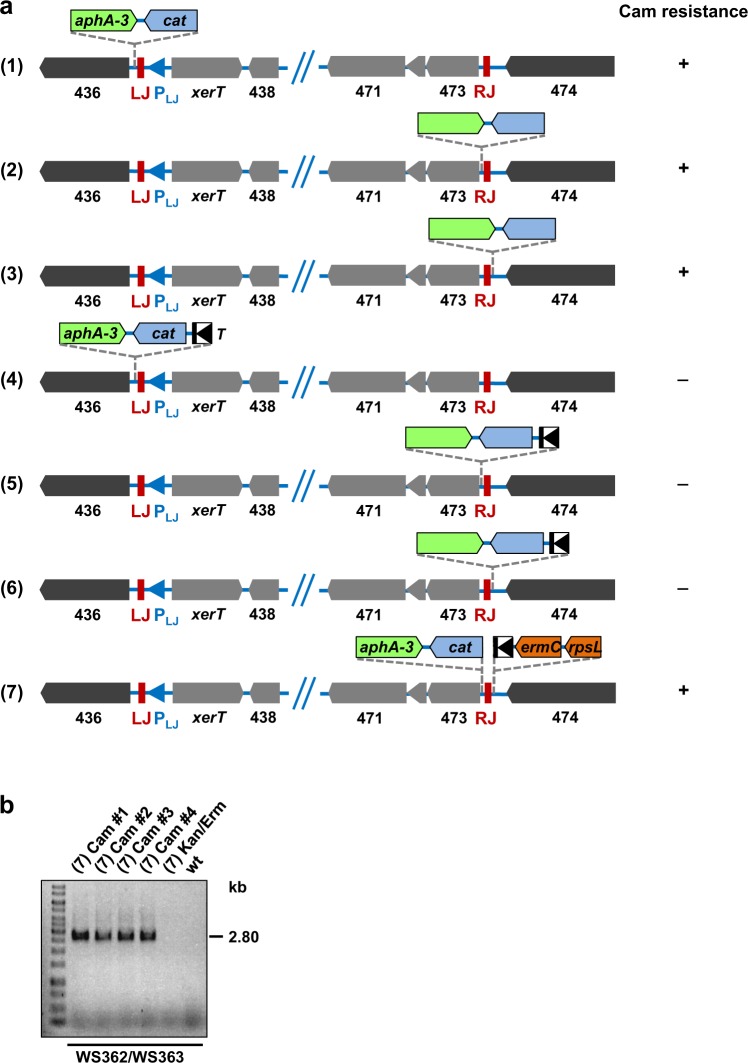
Demonstration of a circularization-dependent promoter activity. (**a**) Different insertions of an *aphA*-3 gene together with a promoterless *cat* gene close to LJ or RJ of ICE*Hptfs4*, or with an additional T-HP1395 terminator (T) upstream of the *cat* gene, were generated in strain P12, resulting in the indicated arrangements after selection for kanamycin or erythromycin resistance (see Materials and Methods for details). Subsequently, kanamycin- or erythromycin-resistant transformants were plated on chloramphenicol-containing media. A (+) sign indicates growth of colonies on chloramphenicol plates, and a (−) sign indicates no growth. (**b**) Genomic DNA preparations of chloramphenicol-selected colonies or non-selected (Kan/Erm) bacteria of mutant (7), or of P12 wild-type, were analysed by PCR with primer pair WS362/WS363 (refer to Fig. 3 for primer binding sites).

To check whether the *xerT* upstream region contains such a promoter activity, we engineered a double gene cassette containing a promoterless *cat* gene and a promoter-containing *aphA*-3 gene for selection. When this cassette was inserted into the P12 chromosome immediately outside the ICE*Hptfs4* LJ (Fig. 4a; construct 1), the resulting clones selected on kanamycin plates had also acquired a resistance to chloramphenicol, suggesting that a promoter activity reading outwards of LJ exists. Inserting the same cassette on either side of the right junction (RJ) (Fig. 4a; constructs 2 and 3) also resulted in chloramphenicol-resistant transformants, indicating another promoter activity outside the ICE*Hptfs4* RJ. Since ICE excision could not be determined in this way, we additionally introduced the T-HP1395 terminator^29^ upstream of the integrated *cat* cassettes and checked for chloramphenicol resistance, as before. None of the corresponding transformants was chloramphenicol-resistant, showing that integration of the terminator effectively precluded transcription from the different promoters (Fig. 4a; constructs 4–6). However, when T-HP1395 was placed immediately outside RJ, and the promoterless *cat*-*aphA-3* cassette close to RJ within ICE*Hptfs4*, we observed single resistant colonies after restreaking the transformants on chloramphenicol (Fig. 4a; construct 7). These colonies were further characterized by PCR amplification of the LJ/RJ annealing region (Figs 3 and 4b). PCR products of the expected sizes were obtained for all chloramphenicol-selected clones, but not for non-selected bacteria or P12 wild-type, and sequencing confirmed the correct recombination of ICE*Hptfs4* to a circular form (data not shown). These results strongly suggest that a promoter activity transcribing outwards of the ICE LJ (henceforth termed P_LJ_) is relocated upstream of *hpp12_473* upon ICE excision and circularization.

### Selection for ICE*Hptfs4* excision does not result in higher transfer rates

**Figure 5:**
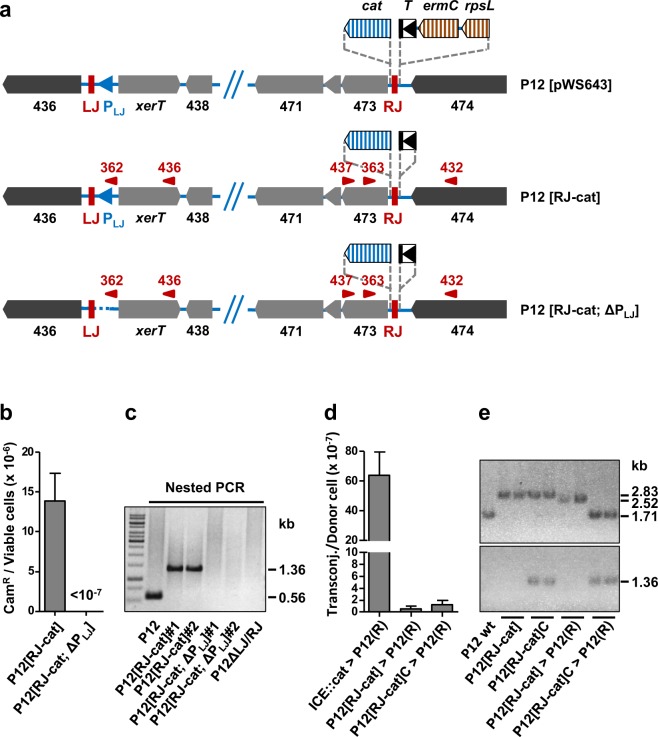
ICE*Hptfs4* excision rates and transfer efficiencies. (**a**) Generation of marker-free chloramphenicol-selectable strains using the *rpsL-erm* counterselection strategy. First, a combination of an *rpsL-ermC*-T-HP1395 cassette outside of ICE*Hptfs4* and the promoterless *cat* cassette within ICE*Hptfs4* (plasmid pWS643) was introduced into a streptomycin-resistant P12 strain by selection for erythromycin resistance. In a second step, the *rpsL-ermC* marker cassette was removed by transformation with plasmid pWS649 lacking the *rpsL-ermC* markers, and selection for streptomycin resistance (resulting in strain P12 [RJ-cat]). Correct insertion of the terminator and the *cat* cassette was confirmed by sequencing. In a similar way, strain P12 [RJ-cat; ΔP_LJ_], additionally lacking the putative P_LJ_ promoter activity upstream of *xerT*, was constructed by two consecutive transformations from P12 [RJ-cat]. (**b**) Excision rates of ICE*Hptfs4* from the chromosome was determined in P12 [RJ-cat] and P12 [RJ-cat; ΔP_LJ_] by plating serial dilutions on non-selective and on chloramphenicol-containing plates, respectively. Data represent average values from six independent experiments with standard errors of the mean. (**c**) Nested PCR with primer pairs WS436/WS437, and subsequently WS362/WS363, was performed from genomic DNA of the indicated strains. (**d**) Chloramphenicol-selected (P12 [RJ-cat]C) and non-selected colonies of P12 [RJ-cat] were provided with a Δ*recA::ermC* mutation and subsequently used as donor strains for mating experiments with the recipient P12(R) (Δ*moeB::aphA*-3). Transfer frequencies are indicated as chloramphenicol/ kanamycin double-resistant clones per viable donor cell. Data represent average values from at least three independent experiments including standard errors of the mean (n = 16, 3, and 3 from left to right). (**e**) Two clones each of strain P12 [RJ-cat] grown without chloramphenicol selection, or with chloramphenicol selection, and transconjugants obtained by mating either non-selected or chloramphenicol-selected P12 [RJ-cat] with P12(R) were characterized by PCR for the presence and properties of the ICE*Hptfs4* RJ region (primers WS432/WS437; upper panel), and for the circular product (primers WS362/WS363; lower panel). A P12 wild-type strain was used as a control. Sizes of the resulting PCR products are indicated on the right (2.83 kb: RJ with *cat* and T-HP1395; 2.52 kb: RJ with *cat*, but without T-HP1395; 1.71 kb: RJ without *cat* and T-HP1395; 1.36 kb: circular form including *cat*).

To estimate the frequency of ICE*Hptfs4* excision and to determine transfer rates from excision-selected bacteria, we generated, using the *rpsL-erm* counterselection strategy, a mutant strain harboring the promoterless *cat* cassette and the T-HP1395 terminator on either side of the ICE*Hptfs4* RJ, as above, but without any further resistance genes (Fig. 5a; P12 [RJ-cat]). Serial dilutions of this mutant were plated on chloramphenicol plates, or on plates without antibiotics, and the excision rate (chloramphenicol-resistant colonies per viable bacterial cells) was calculated from the colony numbers on the respective plates as approximately 1.39 ± 0.34 × 10^−5^ excision events per bacterial cell (Fig. 5b). In order to confirm the contribution of the putative P_LJ_ promoter activity, we deleted this promoter region (nucleotides 61–270 of ICE*Hptfs4* counted from LJ) in P12 [RJ-cat], again using the *rpsL-erm* counterselection strategy (Fig. 5a; P12 [RJ-cat; ΔP_LJ_]). Selection of two independent P12 [RJ-cat; ΔP_LJ_] clones on chloramphenicol plates did not result in resistant colonies, supporting the conclusion that chloramphenicol resistance of P12 [RJ-cat] was due to excision and P_LJ_ promoter relocation (Fig. 5b). Interestingly, these two P12 [RJ-cat; ΔP_LJ_] clones did not contain an ICE excision product in nested PCR analysis, while the wild-type and two P12 [RJ-cat] clones (prior to chloramphenicol selection) did (Fig. 5c), indicating that the P_LJ_ promoter region is also required for excision itself.

Next, we provided both the unselected and chloramphenicol-selected P12 [RJ-cat] mutants with a *recA* deletion and used the obtained mutants as donor strains for mating experiments. Surprisingly, double-resistant colonies were obtained in both cases at frequencies in the range of only 10^−7^ per donor cell, which corresponds to a reduction of about 50-fold in comparison to wild-type donors (Fig. 5d). Thus, selection for excision, using P_LJ_ and the promoterless *cat* cassette, does not increase transfer efficiency of ICE*Hptfs4*, arguing against circular ICE molecules as necessary transfer intermediates. PCR analysis of chloramphenicol-selected P12 [RJ-cat] clones confirmed the appearance of a circular form, whereas non-selected P12 [RJ-cat] clones did not contain sufficient levels of the circular form to be detected (Fig. 5e). Interestingly, however, both the non-selected and selected clones contained the *cat* insertion close to RJ, which means that the *cat* cassette had been duplicated in the chloramphenicol-selected clones (see below). Transconjugants derived from non-selected P12 [RJ-cat] donor strains had obtained the *cat* cassette close to RJ and did not show the “circular” genotype, but had lost the terminator, which explains *cat* gene expression. In contrast, transconjugants derived from chloramphenicol-selected donor strains had obtained the “circular” genotype, but not the *cat* insertion close to RJ (Fig. 5e), which strongly indicates that either a stable circular product with the *cat* cassette had been transferred, or a chromosomal fragment resulting from atypical integration (e.g., non-resolved single crossing-over).

### The excised form of ICE*Hptfs4* is not replicative

**Figure 6:**
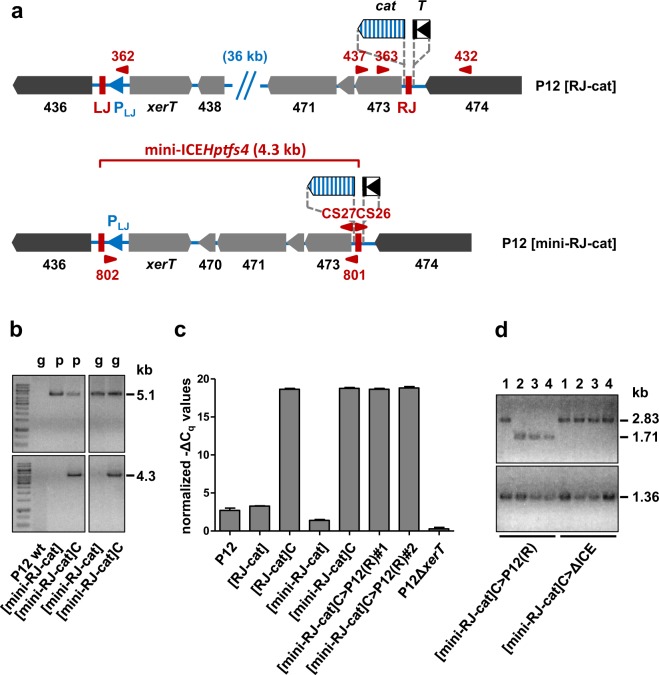
Construction and evaluation of a mini-ICE*Hptfs4* derivative. (**a**) The mini-ICE*Hptfs4* variant (P12 [mini-RJ-cat]) was generated by marker-free deletion of genes 438 to 469 in a strain containing the P_LJ_-activatable *cat* cassette. (**b**) The correct deletion of the ICE*Hptfs4* mid region was confirmed by PCR from genomic (g) or plasmid (p) DNA preparations with the ICE-spanning primer pair WS801/WS802 (upper panels), and rejoining of LJ and RJ after chloramphenicol selection (P12 [mini-RJ-cat]C) was examined by PCR with primers CS26 and CS27 (lower panels). The left and right panels were cropped from single gels; the corresponding full-length gels are presented in Fig. S5. (**c**) Equal amounts of genomic DNA of the indicated strains were analysed by qPCR with primers WS807/WS808 and probe WS809 (amplifying LJ/RJ-rejoined sequences; Fig. S3a), and the resulting C_q_ values were normalized to flanking regions as described in Materials and Methods. The indicated values represent average −ΔC_q_ values from four independent qPCR measurements with standard deviations of the mean. (**d**) Genotypes of transconjugants from the P12 [mini-RJ-cat]C donor to P12 or ΔICE recipients were analysed by PCR with primers WS432/WS437 (upper panel) and WS362/WS363 (lower panel).

Since chloramphenicol-selected ICE*Hptfs4* forms are not transferred at higher rates than integrated forms, we asked whether the excised products are stable, i.e. whether the circular form is able to replicate. Using the *rpsL-erm* counterselection system, we generated a truncated version of the ICE (mini-ICE*Hptfs4*), comprising only *xerT* as well as four genes close to RJ, and provided it with a promoterless *cat* cassette as before (Fig. 6a). Selection of the corresponding strain (P12 [mini-RJ-cat]) on chloramphenicol resulted in appearance of single colonies at a slightly reduced rate in comparison to P12 [RJ-cat] (2.8 × 10^−6^ per viable cell). In accordance with the results described above, these colonies were positive for the circularization PCR (Fig. 6b), while non-selected P12 [mini-RJ-cat] was not, suggesting that mini-ICE*Hptfs4* forms stable circular products (or atypical integration products) upon chloramphenicol selection. However, although PCR reactions were also positive for DNA obtained by plasmid purification (Fig. 6b), we were unable to detect circular DNA molecules of the expected size (5.1 kb) directly.

In order to characterize the excision event further, we set up a qPCR protocol for quantification of excised fragments, using outward-facing primers and normalization to the ICE*Hptfs4* flanking regions (Fig. S3a; see Materials and Methods for details). With this protocol, low amounts of circular products were found for P12 wild-type (data not shown) or the non-selected strains P12 [RJ-cat] and P12 [mini-RJ-cat] (Fig. S3b), and high amounts for chloramphenicol-selected colonies of the latter two strains (Fig. S3b). Quantification demonstrated average differences of 15.4 ± 0.3 and 17.4 ± 0.4 C_q_ values for P12 [RJ-cat] vs. P12 [RJ-cat]C, or P12 [mini-RJ-cat] vs. P12 [mini-RJ-cat]C, corresponding to 40,000-fold or 170,000-fold increases after chloramphenicol selection, respectively (Fig. 6c). After introducing a *recA* mutation into P12 [mini-RJ-cat]C and performing mating experiments, we obtained transconjugants into wild-type recipients at similar rates as with the P12 [RJ-cat]C donors (data not shown). All transconjugant clones had acquired the “circular” genotype, as shown by qPCR or classical PCR, but only some of them had integrated the *cat* cassette close to RJ (Fig. 6c,d). To identify alternative localizations of the *cat* cassette, we analysed ICE*Hptfs4* in selected and non-selected P12 [mini-RJ-cat] as well as in two transconjugant strains by Southern blot. The results clearly show that chloramphenicol selection of P12 [mini-RJ-cat] resulted in duplication of the *cat* cassette (Fig. S4a,b), which is consistent with either stable excision of mini-ICE*Hptfs4*, or tandem mini-ICE insertion after temporary excision (Fig. S4a). However, only the second possibility is able to explain the transfer of both *cat* copies in one transconjugant, and only one *cat* copy in other transconjugants (see Fig. S4 for details). Double integration of mini-ICE*Hptfs4* in the P12 [mini-RJ-cat]C clones was confirmed by detection of a PCR product spanning both ICE copies, and by sequencing a joined LJ/RJ region on this PCR product (data not shown). Independently of the ICE part transferred, all transconjugants had the same levels of rejoined LJ/RJ regions (Fig. 6c, and data not shown), strongly suggesting that each contained a single, chromosomally integrated copy of this rejoined junction. These data, together with the identical behavior of chloramphenicol-selected P12 [RJ-cat] (containing the entire element), demonstrate that the circular form of ICE*Hptfs4* does not replicate, and that selection for excision results in inaccurate (double) integration instead. Moreover, this tandem integration of two ICE*Hptfs4* copies shows that canonical integration by site-specific recombination is functional.

### ICE*Hptfs4* transfer does not require excision

**Figure 7:**
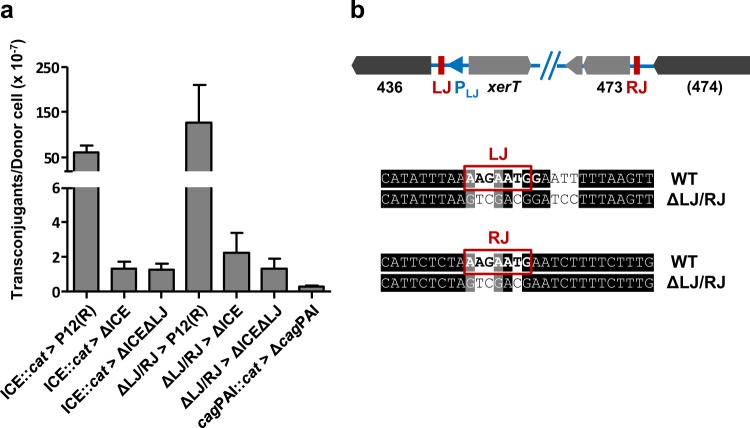
Dispensability of junction sequences for transfer. (**a**) Transfer rates between the indicated donor and recipient strains lacking LJ and/or RJ sequence motifs, or from a donor strain with a cat insertion in the *cag* pathogenicity island (*cag*PAI*::cat*) to a recipient strain lacking the *cag*PAI. Data represent average values from at least three independent experiments including standard errors of the mean (from left to right: n = 16, 14, 9, 3, 3, 3, 3). (**b**) Genotype of the ΔLJ/RJ donor strain lacking both junctional motifs after marker-free deletion.

Although the results described above do not exclude transfer and site-specific integration of the complete ICE*Hptfs4*, the failure of the chloramphenicol-selected donor strains to transfer their marker at increased rates suggests that homologous recombination is more efficient than site-specific recombination, and that excision is not a prerequisite for transfer. If the complete ICE can be integrated by homologous recombination, the presence of the AAGAATG integration motifs should not be required either. In order to examine this hypothesis, we generated a marker-free ICE*Hptfs4* deletion mutant in which the cognate AAGAATG motif was removed as well (Fig. S1), and provided this mutant with a kanamycin resistance. Transfer experiments from donor strain P12 [ICE*Hptfs4::cat*] to this recipient strain resulted in double-resistant clones at a rate similar to that obtained with the ICE*Hptfs4* mutant which still contains the AAGAATG motif (1.1 × 10^−7^ vs. 1.0 × 10^−7^; Fig. 7a), demonstrating that the junction motif is indeed not required for integration, and that transfer under our conditions probably involves homologous recombination. Sequencing of the LJ and RJ regions of the resulting colonies confirmed that the complete island including both junctional motifs had been transferred and integrated (data not shown).

In order to examine whether excision of ICE*Hptfs4* is required in the donor cell, we first performed mating experiments from a donor strain lacking the *xerT* gene, to a recipient strain lacking ICE*Hptfs4* and the AAGAATG integration motif. Again, we found no significant differences between transconjugant rates obtained with an AAGAATG-deleted versus an AAGAATG-containing recipient strain, or obtained with a *xerT* mutant versus a wild-type donor strain (Fig. S2). As a further approach, we generated a mutant in which *xerT* is still present, whereas the LJ and RJ motifs are converted to arbitrary sequences (Fig. 7b). Nested PCR analysis confirmed that this mutant does not excise ICE*Hptfs4* from the chromosome (Fig. 5c). When it was provided with a chloramphenicol resistance and used as a donor strain in mating experiments with wild-type or ICE-deleted recipients, there was again no substantial difference to the wild-type donor with respect to transfer rates (Fig. 7a). Taken together, these data clearly show that excision, and thus probably site-specific recombination, is required neither in the donor, nor in the recipient strain for ICE transfer.

Given that ICE*Hptfs4* transfer is independent of the left and right junction motifs, we finally asked whether transfer of other chromosomal DNA fragments of similar size can occur under these conditions. To examine this, we generated a donor strain with a *cat* gene inserted in a central region of the 37.4 kb *cag* pathogenicity island (*cag*PAI), and a recipient strain lacking the *cag*PAI (Table S1). Mating experiments between these strains resulted indeed in double-resistant clones, albeit at slightly lower rates than for ICE*Hptfs4* to an ICE-negative recipient (3.1 × 10^−8^; Fig. 7a). PCR analysis of these clones confirmed transfer of the entire *cag*PAI (data not shown). Because of the homeologous *cag*PAI flanking regions (Table S1), a recombination breakpoint located 300–350 bp outside the *cag*PAI left junction could be identified in one clone by sequence analysis (data not shown), confirming that the *cag*PAI was inserted in the recipient by homologous recombination.

## Discussion

The concept of integrating conjugative elements, or conjugative transposons, as carriers of accessory genetic material that can rapidly be spread within bacterial populations, is well-established and has been supported in several studies, albeit from a limited number of model systems^2^. For *H*. *pylori*, data obtained by comparing ICE integration sites in different strains strongly suggested that the canonical ICE transfer mechanisms are functional in these bacteria and might be important drivers of strain evolution^12^. However, in this bacterial species with its highly efficient horizontal gene transfer by various pathways including transformation^10^, and also highly efficient homologous recombination systems^27^, canonical ICE transfer mechanisms are clearly superimposed by these more rapid processes.

We have shown in this study that two different strains harboring the same type of ICE are prone to exchange of small ICE fragments, with the potential to form mosaics of ICE subtypes. These results are reminiscent of the outcome observed in natural transformation-driven processes^21,23,30^, with the main difference that our mating experiments were performed in the presence of DNase I, which rules out classical transformation, but leaves recombination mechanisms intact. This suggests that homologous recombination of smaller fragments is favored over site-specific recombination, and the strict requirement of RecA in the recipient strains supports this conclusion. Surprisingly, however, the inner membrane DNA transport protein ComEC^8^ is strictly required in the recipient cells as well. Together with the supportive roles of the outer membrane-associated ComB proteins^7,8^ and of the transformation-associated RecA loader protein DprA^31^, this indicates that the standard DNA uptake system is utilized in this DNase-resistant transfer process. In the same line, we have reported previously that the ComB transformation system also contributes, in the recipient, strongly to transfer efficiency of *H*. *pylori* plasmids under DNase I treatment^24^. This can be explained either by assuming that DNA transfer predominantly occurs by transformation and cannot be prevented by extracellular DNase, or that conjugation between *H*. *pylori* cells takes advantage of the ComB-ComEC system for DNA uptake into the recipient. We consider the first possibility rather unlikely as the sole explanation for several reasons. First, control experiments in which the recipients were incubated with donor strain DNA instead of the strains themselves were clearly susceptible to DNase treatment (data not shown). Second, when the mating experiments were performed in the absence of DNase I, transfer rates were increased by roughly two orders of magnitude for both plasmid^24^ and ICE transfer (data not shown). Third, there seems to be, at least to some degree, a dependence of transfer rates on type IV secretion system genes in the donor (Fig. 2)^13^, arguing for a certain contribution of a conjugation-like process. Fourth, ICE transfer is still possible with a *comB* deletion in the recipient strain, which renders this strain fully transformation-deficient. Remarkably, the reduction of transfer efficiency from the wild-type donor to the Δ*comB* recipient strain is much stronger as from the conjugation-deficient (ΔICEmid) donor to the wild-type strain, suggesting a convergence of different pathways into the ComB, and subsequently the ComEC, pores. One possible explanation for *comB*-independent (or also conjugation-independent) transfer would be a close contact between donor and recipient cells in the mating experiments, which might facilitate DNA transfer in a DNase-insensitive manner. This has been discussed previously as ADR (alternative DNase-resistent) transfer in the case of plasmid conjugation^24^. Nevertheless, it is possible that transformation is responsible for a major part of the *comB*-dependent transfer events observed here, with DNA provided by the donor bacteria in a state protected from DNase degradation. Other ways to reduce natural transformation, such as decreasing the extracellular pH^9^, might be applied to clarify this point in future studies.

Further support for a non-classical ICE transfer mechanism comes from the observation that excision of ICE*Hptfs4* from the chromosome, and thus generation of a circular ICE intermediate, is not a prerequisite for transfer. Both a deletion of the *xerT* gene, or mutation of the integration site, result in a loss of ICE excision, but not in a reduction of ICE transfer, even under conditions where full-length ICE*Hptfs4* is transferred. This strongly suggests that a pathway for mobilization of chromosomal DNA fragments must exist, a conclusion that is also supported by an early report of antibiotic resistance marker transfer between *H*. *pylori* strains^32^. Currently, it is not clear whether ICE DNA can be mobilized directly from chromosomal *oriT* sites by a rolling circle replication mechanism, or which other mechanisms might be involved. Rolling circle replication is usually assumed to occur from circular ICE intermediates^2^, and two possible nicking sites for the ICE*Hptfs4*-encoded relaxase HPP12_451/Rlx2 ((T/C)ATCCTG(C/T)) have already been identified in the ICE*Hptfs4* sequence, as well as in a few other chromosomal locations^33^. Since one HPP12_451/Rlx2 nicking site was identified in ICE*Hptfs3*, ICE*Hptfs3* and ICE*Hptfs4* relaxases (Rlx1/Rlx2)^34^ might be able to replace each other, so that Rlx1 (HPP12_1353) might be involved in ICE*Hptfs4* relaxation as well. In the same context, we cannot rule out that other ICE*Hptfs3* genes are able to take over corresponding functions in the ICE*Hptfs4* gene knockout strains, but it should be noted that strain P12 harbors a truncated ICE*Hptfs3* variant lacking a *virB6* ortholog^13^. In any case, mobilization is clearly RecA-independent, since all donor strains were *recA*-deficient.

Transfer of full-length ICE*Hptfs4* furthermore shows that DNA fragments with a size of more than 40 kb can be recombined into the recipient chromosome, which is not commonly observed for DNA fragments integrated after transformation^23^. The reduced efficiency of full-length ICE integration can thus easily be explained by the size of the fragment to be exchanged, as also evident in the case of the ΔICEmid donor strain, where a fragment of only 5 kb needs to be recombined in the ΔICE recipient strain. Consistent with the finding that large heterologous DNA fragments are prone to degradation by restriction enzymes, while genomic integration of homeologous DNA is permitted^23^, we were able to show transfer of full-length ICE only in a homologous system, but not in a homeologous system, where shorter fragments were exchanged. Nevertheless, site-specific integration of ICE*Hptfs4* via the canonical mechanism must have occurred as well during *H*. *pylori* evolution, since homologous recombination alone cannot explain ICE integration in many chromosomal locations, as observed in strains from different *H*. *pylori* populations^12^. In comparison to the recombination events described here, however, such a canonical integration seems to be rare. Therefore, we expect that coinfection with different *H*. *pylori* strains will frequently lead to reshaping pre-existing ICEs by gene transfer and homologous recombination, or even to integration of full-length ICEs at predefined genomic positions (similar to integration of the *cag*PAI, for which we could show homologous recombination), but only rarely to *de novo* ICE integration by site-specific recombination.

Although ICE*Hptfs4* excision is not required for its transfer, we show that excision constitutively occurs at a rather low rate (in the order of 10^−5^), and that it depends on *xerT*, the AAGAATG integration sequence, and the *xerT* upstream region. Excision of ICEs can be replicative, generating a second, circular copy of the element, but in most cases, excised forms do not replicate^35^. We did not find any evidence for stable excision of ICE*Hptfs4* from the chromosome. Thus, we were unable to isolate circular forms using plasmid preparation protocols. Even an enforced excision of the chloramphenicol-selectable ICE variants did not result in stable circular products. Instead, tandem ICE*Hptfs4* integration by site-specific recombination seems to be the preferred way of dealing with the selective pressure. Remarkably, the chloramphenicol-resistant clones did not revert to the wild-type arrangement when selection was terminated (data not shown), suggesting that this tandem integration is not particularly unstable.

An intriguing feature of ICE*Hptfs4* is that its excision involves relocation of a promoter, with subsequent transcription of genes adjacent to the ICE RJ. This represents one possibility for achieving an excision-dependent switch in the ICE transcriptional profile. It is not clear how many genes are expressed by the relocated promoter; in principle, transcription could go as far as the coupling protein gene (*virD4*/*hpp12_454*) and include almost all type IV secretion genes, similar to the situation in Tn916^36^. On the other hand, an independent transcriptional start site has previously been identified upstream of the *hpp12_471* ortholog *hp0444*^28^, and in an ICE*Hptfs4b* variant, this gene is indeed co-transcribed with several *virB* orthologs^37^. In any case, transcription of *hpp12_473* and *hpp12_472* is very likely switched on in the excised version of ICE*Hptfs4* only. These genes are well conserved in ICE*Hptfs4* elements, although their functions are currently unclear. The principle of a terminal ICE gene which is put under the control of a promoter upon circularization has been described previously for other ICE elements, for example as a means to express the transfer genes^36^, or as a mechanism for ICE maintenance^38^. Generally, ICE activation is subject to complex regulatory networks, which respond to conditions such as DNA damage, cell density, nutrient deprivation, or the presence of antibiotics^35^. Although several ICE*Hptfs4* genes have been reported to be upregulated at pH 5 or upon contact with eukaryotic cells^37^, it is currently unclear how ICE*Hptfs4* activation is regulated. Introduction of a chloramphenicol resistance gene, as in P12 [RJ-cat] or P12 [mini-RJ-cat], and selection for ICE excision simulates in this regard constitutive activation of the ICE, which is generally considered to be deleterious to the host^1^. As discussed above, ICE*Hptfs4* evades this constitutive excision by tandem integration, which does not change the location of the P_LJ_ promoter in comparison to the circular form. Theoretically, site-specific integration into any other permissive AAGAATG motif located in proximity to an appropriate promoter would be possible as well, but was not observed. In any case, tandem integration is most likely a consequence of the inability of circularized ICE*Hptfs4* to replicate.

In conclusion, despite the fact that ICE*Hptfs3* and ICE*Hptfs4* possess all canonical ICE features and have evolved to integrate into many possible chromosomal sites, they are subject to horizontal transfer and integration mechanisms by different pathways, as *H*. *pylori* plasmids and possibly other chromosomal regions are. Even in an environment containing DNases, these pathways are much more efficient than the classical ICE transfer mechanisms involving conjugation and site-specific integration. Site-specific excision is always detectable in a fraction of the bacterial population, suggesting that rapid adaptation to changing environmental conditions is occasionally required. It will be intriguing to identify the environmental cues that lead to ICE*Hptfs4* activation, and thus to better understand the functions of these ancient genome islands.

## Materials and Methods

### Bacterial strains and culture conditions

*H*. *pylori* strains were grown on GC agar plates (Oxoid) supplemented with vitamin mix (1%) and horse serum (8%) (serum plates) and cultured for 16 to 48 h in a microaerobic atmosphere (85% N_2_, 10% CO_2_, 5%O_2_) at 37 °C. *Escherichia coli* strains were grown on Luria–Bertani (LB) agar plates supplemented with antibiotics, as appropriate. Plasmids were introduced into *H*. *pylori* by transformation as described previously^39^, and transformants were selected on serum plates containing 6 mg/l chloramphenicol, 8 mg/l kanamycin, 10 mg/l erythromycin, or 250 mg/l streptomycin, as appropriate.

### Plasmid constructs

All standard PCR amplification, cloning and DNA analysis procedures were performed according to^40^. For generation of an ICE*Hptfs4* deletion strain (Table S1), plasmids pWS377, and pWS415 were constructed by cloning a PCR product amplified with primers WS429 and WS436 (Table S2) together with a *cat* cassette (pWS377), or an *rpsL-erm* cassette (pWS415), into the *Xho*I and *Bam*HI sites of the *tfs4*/*virD2* deletion plasmid pSR31^24^. For plasmids pWS328 and pWS329, ICE*Hptfs4* flanking regions from strain P12 were amplified by PCR using primer pairs WS429/WS430, and WS431/WS432, respectively, and cloned with (pWS328) or without (pWS329) an *rpsL-erm* cassette into the *Xho*I and *Not*I sites of pBluescript II SK. Additional deletion of the remaining LJ sequence motif was achieved by inverse PCR from pWS328 with primers WS793/WS794 (plasmid pWS659). Plasmid pWS671, used for generation of P12 [mini-RJ-cat], is an *rpsL-erm* (*Bam*HI) deletion derivative of pWS415. The *comEC* deletion plasmid pSR33 was generated by inverse PCR from a pSMART-HcKan vector containing a P12 chromosomal fragment (HP2kb-02_C16) with primers SR111/SR112, restriction with *Bam*HI and ligation with an *aphA*-3 cassette. Likewise, the *dprA* deletion plasmid pCS5 was constructed using plasmid HP5kb-05_G08 and primers CS6/CS7 (ligation with an *rpsL*-*erm* cassette via *Sal*I and *Bam*HI), and the *virB6* deletion plasmid pBK2 using plasmid HP5kb-05_G24 and primers BK5/BK6 (ligation with *cat* via *Sal*I and *Bam*HI). For introduction of a kanamycin resistance gene into the *moeB* locus, plasmid pSR20^24^ was used. For the construction of *cat* reporter strains, flanking regions were amplified with primer pairs WS429/CS25 (gene 436), CS37/CS27 (gene 473 without RJ), CS37/CS36 (gene 473 with RJ), CS24/WS436 (LJ and gene 437), CS26/CS39 (RJ and gene 474), or CS38/CS39 (gene 474 without RJ). A promoterless, but terminator-containing *cat* cassette was amplified with primers CS23/RH136 from pHel2, restricted with *Xba*I and *Not*I, and combined with *aphA*-3 from plasmid pHel3 (*Sac*I/*Xba*I) to obtain a *Sac*I/*Not*I-restricted *aphA*-3-*cat* fragment. This fragment was subsequently cloned together with the corresponding left and right flanking regions described above (either via *Xho*I/*Sac*I and *Not*I/*Bam*HI, or via *Bam*HI/*Sac*I and *Not*I/*Sac*II) into the respective sites of pBluescript II KS+ (resulting in plasmids pCS11, pCS14 and pCS15; constructs (1)-(3) in Fig. 4a). Alternatively, the T-HP1395 terminator was amplified with primers CS80/CS81 (*Sac*I/*Sal*I) and cloned together with an *rpsL*-*erm* cassette (*Sal*I/*Not*I) and the *cat* cassette (amplified with primers CS23/CS79 or CS23/CS36, and restricted *Xba*I/*Sac*I) between the same flanking regions (resulting in plasmids pCS16-pCS19 (constructs (4)-(7) in Fig. 4a). Plasmid pWS643 was constructed by amplification of the ICE*Hptfs4* right border region with primers WS437/WS785 and cloning into the *Kpn*I and *Pst*I sites of pCS19. Plasmid pWS649, which was used to generate P12 [RJ-cat], was obtained by deletion of *rpsL*-*erm* as a *Bam*HI fragment from pWS643. For deletion of the LJ/RJ regions, plasmid pWS664, which was constructed by inverse PCR from HP2kb-07_P06 with primers WS800/WS801 and religation (via *Sal*I), was transformed into P12 [pWS643], thus deleting the RJ sequence together with the *cat* and T-HP1395 terminator/*rpsL*-*erm* cassettes. Subsequently, the LJ sequence was removed by sequential transformation with plasmids pWS666 and pWS665 (inverse PCR from HP5kb-02_P16 with primers WS802/WS803; religation with or without *rpsL-erm*, respectively). An inverse PCR was furthermore performed from HP5kb-02_P16 with primers WS804/805, and the product was religated after *Sac*I restriction (plasmid pWS667). To delete the promoter region upstream of *xerT*, while keeping the promoterless *cat* cassette close to the RJ, strain P12 [RJ-cat] was sequentially transformed with plasmids pWS666 and pWS667. Insertion of the *cat* cassette into the *cag*PAI was accomplished by transformation with plasmid pWS316, which was generated by inverse PCR with primers WS406 and WS407 from HP2kb-04_P16 and religation with *cat*. Marker-free deletion of the *cag*PAI was achieved by sequential transformation with plasmids pJP44 (*rpsL-erm*) and pJP44^24^. Genomic DNA of *H*. *pylori* strains was extracted using the QIAamp DNA Mini kit (Qiagen), and plasmid DNA was extracted using the Wizard Plus SV Minipreps system (Promega).

### Mating experiments

Mating experiments to determine transfer of ICE*Hptfs4* into recipient strains was performed as previously described for plasmid transfer^24^. Briefly, donor strains were provided with a *cat* cassette in an intergenic region of ICE*Hptfs4* (between *hpp12_453* and *hpp12_454*), and recipient strains with an *aphA-3* cassette in the *moeB* locus. To prevent transfer of chromosomal recipient strain DNA into donor strains, *recA* was deleted in all donor strains with plasmid pDH29 (Δ*recA::erm*). Donor and recipient strains grown on selective plates were each adjusted to a concentration of 1.5·10^7^ cells in a volume of 50 µl *Brucella* broth containing 0.5 mg/ml (>1000 U/ml) DNase I (Roche), and incubated for 30 min at 37 °C and 10% CO_2_. Subsequently, donor and recipient cells were mixed and spotted onto non-selective serum plates, and incubated overnight at 37 °C under microaerobic conditions. Bacteria were resuspended, and serial dilutions were plated on serum plates containing either chloramphenicol (for donor cell counts) or chloramphenicol and kanamycin (for transconjugants). Transfer rates were determined as numbers of transconjugant colonies per donor cfu.

### Quantitative PCR

Genomic DNA of *H*. *pylori* strains was extracted with a QIAamp DNA Mini kit (Qiagen) according to the manufacturer’s instructions, and DNA concentrations were measured with a NanoDrop 2000 spectrophotometer (Thermo Fisher Scientific). Primers and probes for qPCR were designed using Primer Express 3.0 (Thermo Fisher Scientific) for an optimal annealing temperature of 60 °C. Hydrolysis probes were 5′ end-labelled with 6-FAM fluorophores, and 3′ end-labelled with BHQ-1 quenchers. All primers and probes were synthesized by Metabion (Planegg, Germany). Conditions for qPCR were established according to the MIQE guidelines^41^. The qPCR reactions were carried out in a LightCycler 96 Real time PCR instrument (Roche) according to the manufacturer’s instructions. Briefly, reaction mixes were set up with FastStart Essential DNA Probes master mix (Roche) in a volume of 20 µl with 2.5 µl of genomic DNA (adjusted to a concentration of 35 ng/µl), and final concentrations of 0.3 µM primers and 0.25 µM probes. Samples were loaded into 96-well white plates (Roche) and run with a 2-step amplification protocol (95 °C for 15 s, 60 °C for 60 s) for 45 cycles. The quantification cycles and baselines were automatically determined, and results were further analysed using the LightCycler 96 v1.1 software (Roche). For determination of standard curves, PCR products were obtained either from genomic DNA of a P12 [mini-RJ-cat] to P12(R) transconjugant clone with primers WS362/WS363, or from genomic DNA of P12 with primers WS432/WS813, or with primers WS429/WS436, and examined in dilution series with primer/probe sets WS807/WS808/WS809, WS811/WS813/WS812, or WS808/WS810/WS809, respectively. PCR efficiencies were 96%, 108%, and 94%, respectively, with a detection limit of four gene copies for the WS807/808/809 qPCR. Specificity was confirmed by performing qPCR measurements with genomic DNA from the appropriate *H*. *pylori* mutants. For evaluation, the relative quantities of excised products were normalized to C_q_ values obtained either with primers WS811/WS813 and probe WS812 (amplifying the ICE*Hptfs4* right flanking region), or with primers WS808/WS810 and probe WS809 (amplifying the LJ region). For normalization to the right flanking regions, results were calculated as −ΔC_q_ values according to the formula −ΔC_q_ = [C_q,Ref_ (WS811/812/813) −C_q,Exc_ (WS807/808/809)] +ΔC_q,Ctr_, where −ΔC_q,Ctr_. is the average −ΔC_q_ value for a negative control mutant lacking ICE*Hptfs4*.

### Southern Blot

Southern blotting of genomic *H*. *pylori* DNA and hybridizations with PCR fragments as probes were performed using the ECL Direct Nucleic Acid labelling and detection system (GE Healthcare) according to the manufacturer’s protocol, except that 10x SSPE (1.8 M NaCl, 100 mM sodium phosphate pH 7.5, and 10 mM EDTA) was used instead of 10x SSC for blotting. The hybridization buffer contained 0.5 M NaCl, and the primary washing buffer contained 0.5x SSC and 0.4% SDS.

### Statistical analysis

Quantitative data are generally shown as average values from at least three independent experiments with standard errors of the mean, with individual numbers of experiments as indicated. Statistical significance was calculated via Student’s t-test or one-way ANOVA, using the GraphPad Prism software. All indicated p values are two-tailed values.

## Data Availability

The datasets generated during and/or analysed during the current study are available from the corresponding author on reasonable request.

## Supplementary information

Supplementary Data
