# Supplementary material for: Excision and transfer of an integrating and conjugative element in a bacterial species with high recombination efficiency
Source: Sci Rep. 2019 Jun 20;9:8915. doi: 10.1038/s41598-019-45429-z (PMC6586827; doi:10.1038/s41598-019-45429-z)
Supplement: Supplementary file 1 — Supplementary Data [file 41598_2019_45429_MOESM1_ESM.pdf]

Supplementary Data

Excision and transfer of an integrating and conjugative element in a bacterial species with high recombination efficiency

Evelyn Weiss, Carolin Spicher, Rainer Haas and Wolfgang Fischer[*]

Max von Pettenkofer Institute of Hygiene and Medical Microbiology, Faculty of Medicine, LMU Munich, Germany

Supplementary Figures S1-S5

Supplementary Tables S1 and S2

Supplementary Figures

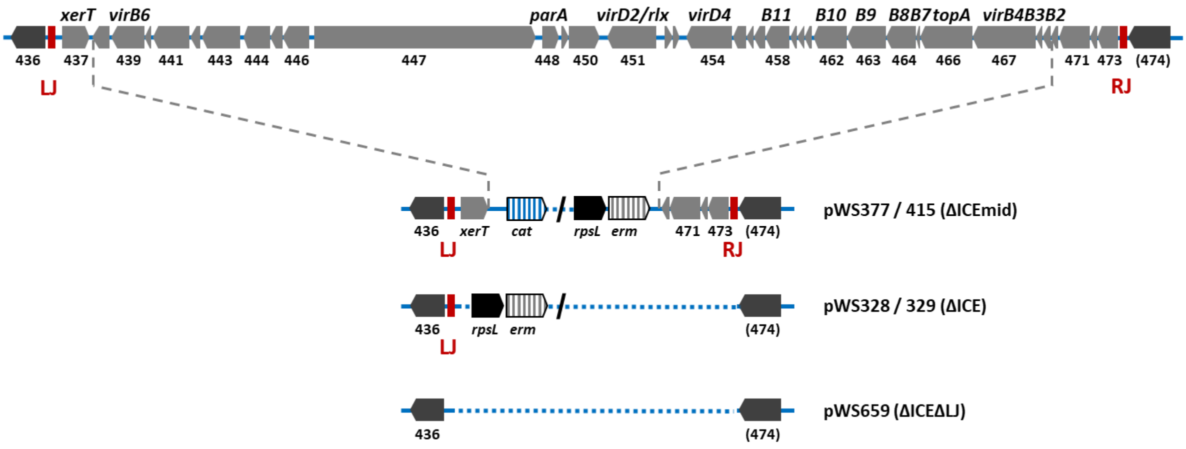

Fig. S1. Construction of ICE*Hptfs4* deletion mutants. For deletion of the major part of ICE*Hptfs4*, left and right flanking regions including *xerT* (*hpp12_437*) and *hpp12_470-473* were cloned together with a *cat* or an *rpsL-ermC* cassette, resulting in plasmids pWS377 and pWS415, respectively. For complete deletion, flanking regions excluding these genes were cloned with an *rpsL-ermC* cassette or without any resistance marker (pWS328 and pWS329). In these strains, one of the junction sequences is still present, as in *H. pylori* strains which do not have an ICE insertion in this site. For plasmid pWS659, this junction sequence was removed as well.

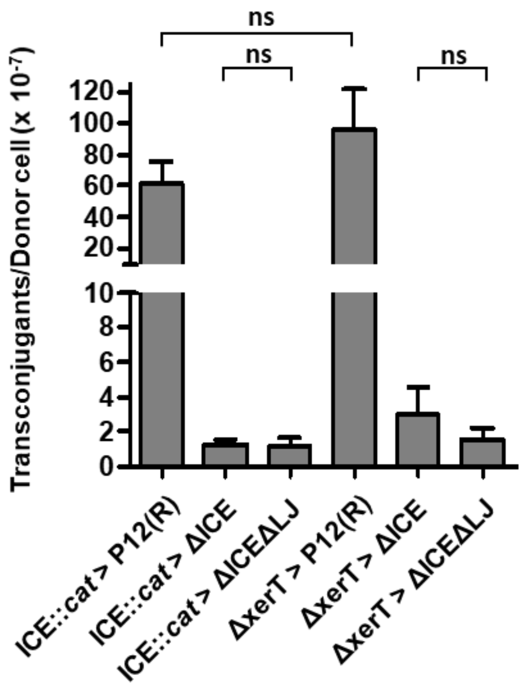

Fig. S2. ICE transfer rates in excision- or integration-deficient donor or recipient mutants. Transfer frequencies are indicated as chloramphenicol/ kanamycin double-resistant clones per viable donor cell. Data represent average values from at least three independent experiments (from left to right, n=16, 14, 6, 5, 6, 3, 7, 5, 3) including standard errors of the mean. ns, no significant difference.

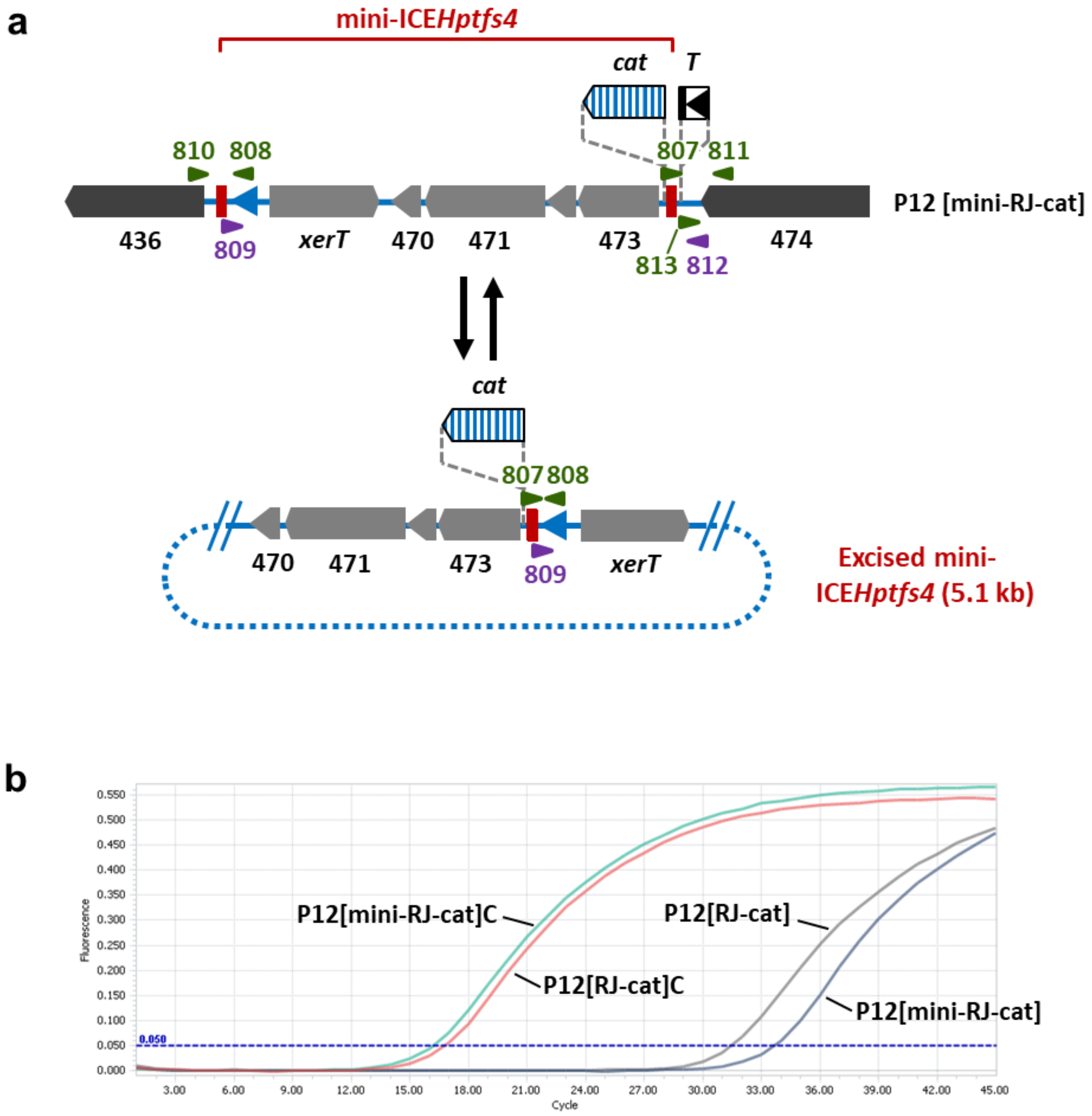

Fig. S3. Quantitative determination of excision by qPCR. (A) Primers (green arrowheads) and probes (purple arrowheads) for LightCycler amplification of circular or integrated forms of ICE*Hptfs4*, as well as of the flanking genomic region, were designed as indicated. (B) Representative amplification curves of excision-selectable strains P12 [RJ-cat] and P12 [mini-RJ-cat] with or without chloramphenicol selection.

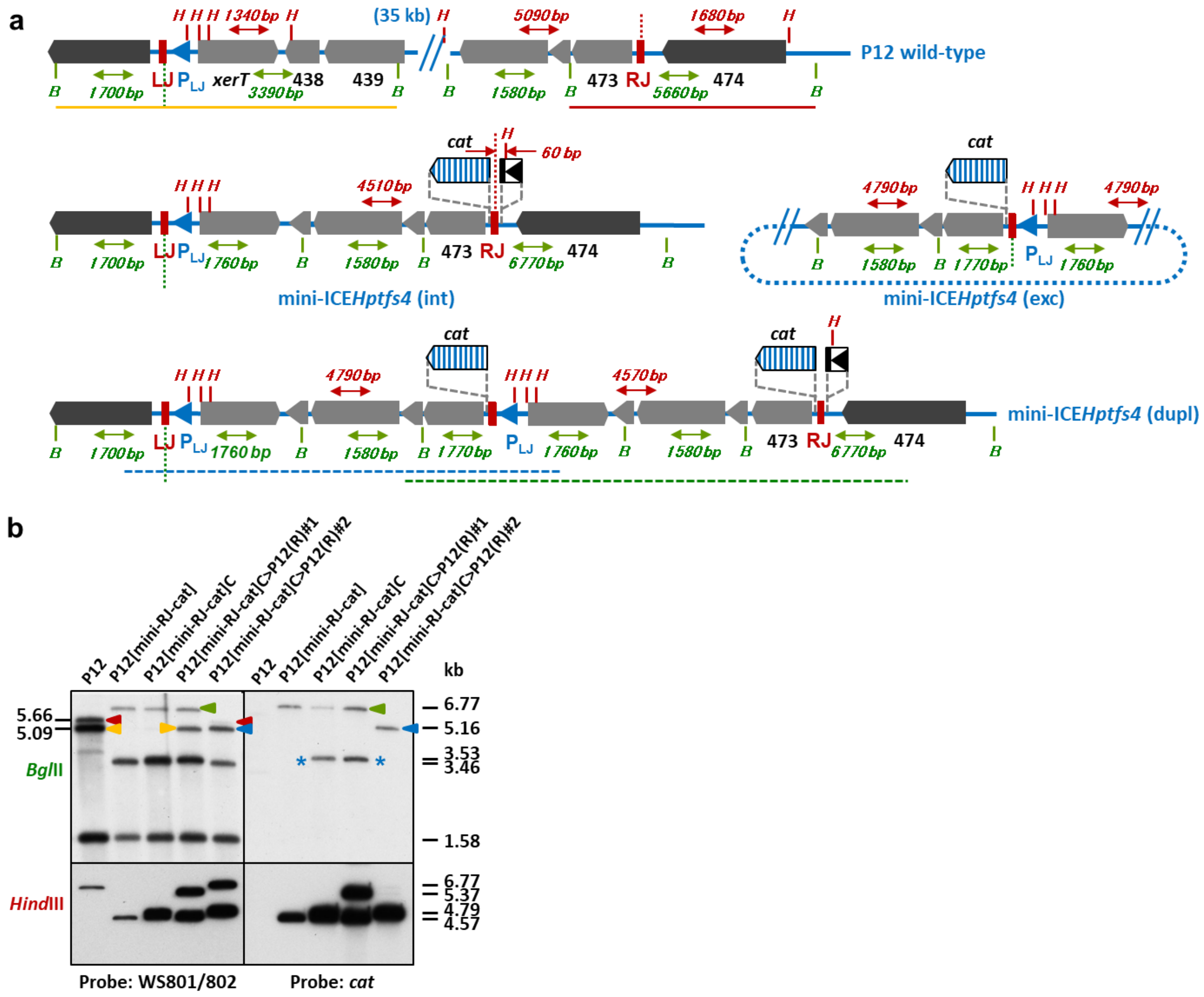

Fig. S4. Southern blot analysis of mini-ICE*Hptfs4* excision and integration after transfer. (a) Schematic maps of ICE*Hptfs4*, and of mini-ICE*Hptfs4* in its integrated, excised and double-integrated forms are shown with locations of *Bgl*II (B) and *Hind*III (H) sites. (b) Southern blots of *Bgl*II- or *Hind*III-digested genomic DNA of the indicated strains developed with probes comprising either mini-ICE*Hptfs4* (WS801/802), or the *cat* cassette only. Additional copies of the *cat* cassette in the chloramphenicol-selected P12 [mini-RJ-cat] strain and in one transconjugant clone are indicated by asterisks. The yellow and red arrowheads indicate 5.09 kb, or 5.66 kb *Bgl*II fragments covering the LJ or RJ in the wild-type, respectively (corresponding to the yellow or red lines in panel a). The green arrowheads (6.77 kb *Bgl*II fragments) correspond to the RJ region of integrated mini-ICE*Hptfs4* including the *cat* cassette, which would be the result of transfer of this region into a wild-type recipient. However, since

both P12 [mini-RJ-cat]C and the first transconjugant clone contain two different copies of the *cat* cassette, the only explanation is that a DNA fragment containing both *cat* copies had been transferred (dashed green line in panel a). The blue arrowheads (5.16 kb *Bgl*II fragments) result from transfer of a LJ region including the *cat* cassette (dashed blue line in panel a) to the wild-type recipient.

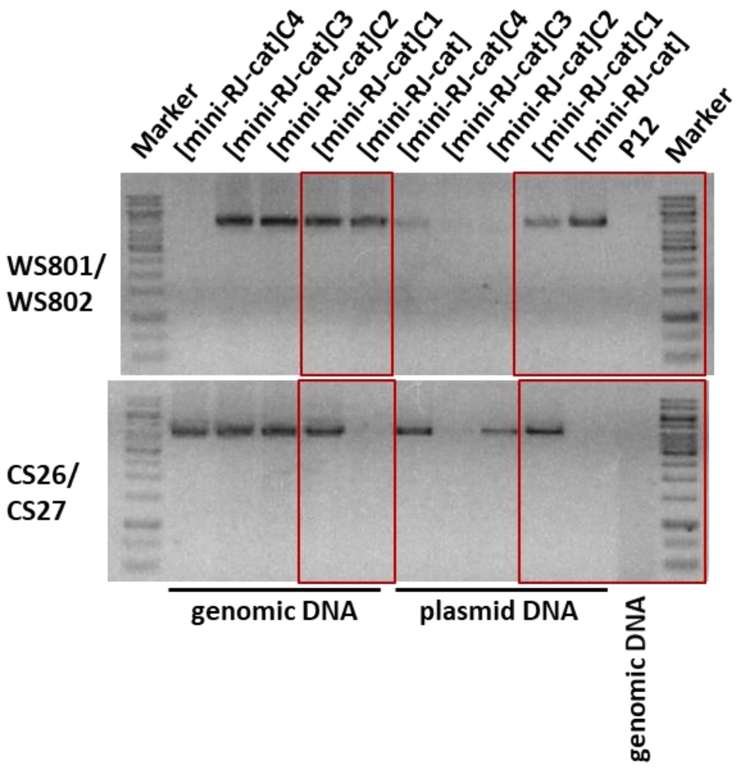

Fig. S5. Uncropped images of the gels shown in Fig. 6b. PCR products obtained with WS801/WS802 or CS26/CS27 primers from either genomic DNA or plasmid preparations of the indicated strains (P12 wild-type, P12 [mini-RJ-cat], or 4 individual clones of P12 [mini-RJ-cat] selected on chloramphenicol plates) were loaded, as indicated. The areas used for generating the cropped images shown in Fig. 6b (after horizontal mirror inversion) are indicated by red boxes.

Supplementary Tables

Table S1. *H. pylori* strains used in this study.

Strain	Genotype or description	Reference
G27	wt	(13)
P12	wt	(21)
P12 ICE::*cat*	Donor; ICE*Hptfs4::cat* (insertion between *hpp12_453* and *hpp12_454*); Δ*recA::erm*	(13)
G27 ICE::*cat*	Donor; ICE*Hptfs4::cat* (insertion between *hpg27_974* and *hpg27_975*); Δ*recA::erm*	This study
P12Δ*xerT*	Δ*xerT::cat;* Δ*recA::erm*	(13)
P12Δ*virB6*	Δ*virB6::cat;* Δ*recA::erm*	This study
P12Δ*virD4*	Δ*virD4::cat;* Δ*recA::erm*	(13)
P12(R)	Recipient; Δ*moeB::aphA-3*	(13)
P12ΔICEmid	Δ*hpp12_438-hpp12_469::cat* (pWS377); Δ*recA::erm*	This study
P12ΔICE	ΔICE*Hptfs4::rpsL-erm;* Δ*moeB::aphA-3*	This study
P12Δ*comB*	Δ*comB7-10::aphA-3*	(7)
P12Δ*comEC*	Δ*comEC::aphA-3*	This study
P12Δ*recA*	Δ*recA::erm*; Δ*moeB::aphA-3*	This study
P12Δ*dprA*	Δ*dprA::rpsL-erm;* Δ*moeB::aphA-3*	This study
P12 [pCS19]	ICE*Hptfs4::cat-aphA-3* (insertion upstream of *hpp12_473*); *rpsL-erm*-T-HP1395 (insertion outside ICE*Hptfs4* RJ)	This study
P12 [pWS643]	ICE*Hptfs4::cat* (insertion upstream of *hpp12_473*); *rpsL-erm*-T-HP1395 (insertion outside ICE*Hptfs4* RJ)	This study
P12 [RJ-cat]	ICE*Hptfs4::cat* (insertion upstream of *hpp12_473*); T-HP1395 (insertion outside ICE*Hptfs4* RJ)	This study
P12 [RJ-cat; ΔP_{LJ}]	ICE*Hptfs4::cat* (insertion upstream of *hpp12_473*); T-HP1395 (insertion outside ICE*Hptfs4* RJ); ΔP_{LJ}	This study
P12 [mini-RJ-cat]	mini-ICE*Hptfs4::cat* (Δ*hpp12_438-hpp12_469* marker-free; *cat* insertion upstream of *hpp12_473*); T-HP1395 (insertion outside ICE*Hptfs4* RJ)	This study
P12ΔICEΔLJ	ΔICE*Hptfs4* (marker-free; pWS659)*;* Δ*moeB::aphA-3*	This study
P12ΔLJ/RJ	marker-free deletion of LJ and RJ (pWS643; pWS664; pWS666; pWS665); ICE*Hptfs4::cat* (insertion between *hpp12_453* and *hpp12_454*); Δ*recA::erm*	This study
P12 *cag*PAI::*cat*	Donor; Δ*cagP::cat*; Δ*recA::erm*	This study
P12ΔPAI(R)	Recipient; Δ*cag*PAI (marker-free, with flanking regions from strain 26695); Δ*moeB::aphA-3*	(24); this study

Table S2. Oligonucleotide primers used in this study.

Primer	Sequence (5’-3’)	Position in P12 genome	Remarks
BK5	CGGGATCCCTCATAGAGTTAGCAAATGATAAAGG	454353-454328	*Bam*HI
BK6	AGTCGTCGACCAAGGCAAGTAGTGGAGCG	455573-455591	*Sal*I
CS6	ATGTCGACTTAGGCAGTATGATCTCTAC	343776-343757	*Sal*I
CS7	CGGGATCCGCATGATTTTAGCGTGCGATG	344583-344602	*Bam*HI
CS23	AGTATCTAGAGATCCACGTTGAAAATCT	*cat* cassette	*Xba*I
CS24	TGACGCGGCCGCACACAAAGACTATATCCGTATC	451987-452008	*Not*I
CS25	ACTAGAGCTCGTCTCAAACGCCTTTATATC	451953-451934	*Sac*I
CS26	AGCTGCGGCCGCAATTCCTAGATATTGATTTCTTAGAG	492717-492742	*Not*I
CS27	GATCGAGCTCGACCATGCAAGAAAGAGTTT	492724-492695	*Sac*I
CS36	GATCGAGCTCGCCTGATTTTGCATGACGAAC	492814-492794	*Sac*I
CS37	GTACGGATCCTCCCATAGCGATTTCAATTTCC	491765-491786	*Bam*HI
CS38	CATGGCGGCCGCGGTTAGGGTTTTCTTGTAAGAC	492845-492866	*Not*I
CS39	CTAGCCGCGGAAAAGGCATCATCTGGCACAC	493761-493741	*Sac*II
CS79	GATCGAGCTCGCTAAGGAAGCTAAAATGGAG	*cat* cassette	*Sac*I
CS80	ACGTGTCGACCTTGGATATAGGGTTTAGGCG	T-HP1395 terminator	*Sal*I
CS81	GATCGAGCTCCACCAAAGCTTTAAGCCAAGC	T-HP1395 terminator	*Sac*I
RH136	ATAAGAATGCGGCCGCTAAATGACTAAGGAAGCTAAA ATGGAG	*cat* cassette	*Not*I
SR111	GATCGGATCCTTTTAGTTTCTTGTCCCTCT	1431301-1431320	*Bam*HI
SR112	GATCGGATCCTAGCGTTACTCCCTAAATTG	1430473-1430454	*Bam*HI
WS362	TATTTTAAGCTTTGAGCTCCC	452307-452287	
WS363	TTTCTTACAGCCTGTGCCACC	492501-492521	
WS406	GCGGATCCCAAAATTGGTTCTTATGATTC	573638-573618	*Bam*HI
WS407	ACGCGTCGACAATCGGTCGTTTCATTTTGG	573960-573979	*Sal*I
WS429	ACCGCTCGAGATTATTGTATTTGCTTAATAGG	451106-451127	*Xho*I
WS430	GCGGATCCGAATTCCATTCTTTTAAATATG	452031-452011	*Bam*HI, *Eco*RI
WS431	ACCGGTCGACGAATTCTTTCTTTGACTTCTTGTTC	492779-492797	*Sal*I, *Eco*RI
WS432	GATCGCGGCCGCTTTATTGTGGACAGGTTGG	493649-493631	*Not*I
WS436	CGGGATCCGAAAATTTAAGTGGTTTGTG	453598-453579	*Bam*HI
WS437	CGGGTACCTTATGATGCTAAAATTTCC	491931-491949	*Kpn*I
WS785	ACCGCTGCAGGACCATGCAAGAAAGAG	492716-492698	*Pst*I
WS793	GAATCTTTTCTTTGACTTCTTG	492773-492794	
WS794	TAAATATGAAGATACGGATATAG	452018-451996	
WS800	ACTGGTCGACGAATCTTTTCTTTGACTTCTTGT	492773-492795	*Sal*I
WS801	ACGTGTCGACTAGAGAATGCAATTATTTTCTAATC	492766-492742	*Sal*I

WS802	ACGTGTCGACGGATCCTTTAAGTTAAATCTAATCTACAAAATG	452032-452058	*Sal*I, *Bam*HI
WS803	ACGTGTCGACTTAAATATGAAGATACGGATATAGTC	452019-451994	*Sal*I
WS804	ACGTGAGCTCTTGGTATAACAACGGAGTAATC	452079-452058	*Sac*I
WS805	GTATTCATGGGAGCTCAAAG	452279-452298	
WS807	AATTCCTAGATATTGATTTCTTAGAGATTAGAAAA	492717-492751	qPCR primer
WS808	TTTTAAGCTTTGAGCTCCCATGA	452305-452283	qPCR primer
WS809	AGCTCACATTCTTTTTCCTGTTTTTCCTTTATCCTTTC	452124-452161	5‘-6-FAM; 3‘-BHQ-1
WS810	TGTTAAACACAAAGACTATATCCGTATCTTC	451981-452011	qPCR primer
WS811	CAGAGATTTATTGAATTTCAGGCGT	492987-492963	qPCR primer
WS812	CAACTCCAATCCAGCCTGATTTTGCATG	492827-492800	5‘-6-FAM; 3‘-BHQ-1
WS813	GAATCTTTTCTTTGACTTCTTGTTCGT	492773-492799	qPCR primer
